# Integration of Genetic and Clinical Risk Factors for Risk Classification of Uveitis in Patients With Juvenile Idiopathic Arthritis

**DOI:** 10.1002/art.42955

**Published:** 2024-09-05

**Authors:** Melissa Tordoff, Samantha L. Smith, Saskia Lawson‐Tovey, Andrew D. Dick, Michael W. Beresford, Athimalaipet V. Ramanan, Kimme L. Hyrich, Andrew P. Morris, Stephen Eyre, Lucy R. Wedderburn, John Bowes, the CLUSTER Consortium, Lucy R. Wedderburn, Zoe Wanstall, Vasiliki Alexiou, Fatjon Dekaj, Bethany R. Jebson, Melissa Kartawinata, Aline Kimonyo, Eileen Hahn, Genevieve Gottschalk, Freya Luling Feilding, Alyssia McNeece, Fatema Merali, Elizabeth Ralph, Emily Robinson, Emma Sumner, Andrew Dick, Michael W. Beresford, Emil Carlsson, Joanna Fairlie, Jenna F. Gritzfeld, Oliver McClurg, Karen Rafferty, Athimalaipet V. Ramanan, Teresa Duerr, Michael Barnes, Sandra Ng, Kimme Hyrich, Stephen Eyre, Soumya Raychaudhuri, Wendy Thomson, John Bowes, Jeronee Jennycloss, Saskia Lawson‐Tovey, Paul Martin, Andrew Morris, Stephanie Shoop‐Worrall, Samantha Smith, Michael Stadler, Damian Tarasek, Melissa Tordoff, Annie Yarwood, Chris Wallace, Wei‐Yu Lin, Nophar Geifman, Sarah Clarke, Thierry Sornasse, Robert J. Benschop, Rona Wang, Daniela Dastros‐Pitei, Sumanta Mukherjee, Michael McLean, Anna Barkaway, Peyman Adjamian, Helen Neale

**Affiliations:** ^1^ The University of Manchester Manchester United Kingdom; ^2^ NIHR Manchester Biomedical Research Centre, Manchester University NHS Foundation Trust Manchester UK; ^3^ University of Bristol, Bristol, UCL Institute of Ophthalmology, London, and Moorfields Eye Hospital London United Kingdom; ^4^ University of Liverpool and Alder Hey Children's NHS Foundation Trust Hospital Liverpool United Kingdom; ^5^ Bristol Royal Hospital for Children and University of Bristol Bristol United Kingdom; ^6^ UCL Great Ormond Street Institute of Child Health, UCL Hospital and Great Ormond Street Hospital, and NIHR Biomedical Research Centre at Great Ormond Street Hospital London United Kingdom

## Abstract

**Objective:**

Juvenile idiopathic arthritis (JIA)–associated uveitis (JIAU) is a serious JIA comorbidity that can result in vision impairment. This study aimed to identify genetic risk factors within the major histocompatibility complex for JIAU and evaluate their contribution for improving risk classification when combined with clinical risk factors.

**Methods:**

Data on single nucleotide polymorphisms, amino acids, and classical HLA alleles were available for 2,497 patients with JIA without uveitis and 579 patients with JIAU (female 2,060, male 1,015). Analysis was restricted to patients with inferred European ancestry. Forward conditional logistic regression identified genetic markers exceeding a Bonferroni‐corrected significance (6 × 10^−6^). Multivariable logistic regression estimated the effects of clinical and genetic risk factors, and a likelihood ratio test calculated the improvement in model fit when adding genetic factors. Uveitis risk classification performance of a model integrating genetic and clinical risk factors was estimated using area under the receiver operator characteristic curve and compared with a model of clinical risk factors alone.

**Results:**

Three genetic risk factors were identified, mapping to *HLA‐DRB1*, *HLA‐DPB1*, and *HLA‐A*. These markers were statistically independent from clinical risk factors and significantly improved the fit of a model when included with clinical risk factors (*P* = 3.3 × 10^−23^). The addition of genetic markers improved the classification of JIAU compared with a model of clinical risk factors alone (area under the curve 0.75 vs 0.71).

**Conclusion:**

Integration of a genetic and clinical risk prediction model outperforms a model based solely on clinical risk factors. Future JIAU risk prediction models should include genetic risk factors.

## INTRODUCTION

Genetic risk factors have the potential to aid classification and prognostic tools to assess risk of complications in rare diseases with the potential benefit of improving early diagnosis and treatment of the disease. Juvenile idiopathic arthritis (JIA)–associated uveitis (JIAU) is a serious complication of JIA, which can lead to loss of sight. JIAU onset in the majority of patients (88.7%) is within 4 years of JIA diagnosis.^1^ Early detection and treatment of JIAU is paramount to avoid ocular inflammation and severe complications that can lead to visual loss.^2^, ^3^, ^4^ The disease affects ~13% of patients, rising to 30% of patients in oligoarticular and rheumatoid factor (RF)–negative polyarticular International League of Associations for Rheumatology (ILAR) JIA subtypes, which makes JIAU the most common extra‐articular manifestation in patients with JIA.^3^, ^5^, ^6^, ^7^

There are a number of JIAU screening guidelines, including the 2006 British Society for Paediatric and Adolescent Rheumatology (BSPAR) guidelines and the 2019 American College of Rheumatology guidelines.^8^, ^9^ Although the current guidelines are comprehensive, expert review of the current screening approaches by the Single Hub and Access Point for Paediatric Rheumatology in Europe initiative considers these to be suboptimal with respect to who should be screened and when screening should occur, resulting in a significant burden to the child and family.^10^ Furthermore, routine examination is challenging in children as complete examination is necessary, which can require anesthesia in some instances.^11^ Screening guidelines use the clinical risk factors of antinuclear antibody (ANA) status, age at onset (AAO) of JIA, and ILAR subtype of JIA. Sex is also considered a clinical risk factor for JIAU because girls and young women are at a greater risk of JIAU development.^12^ However, screening guidelines can differ in their respective recommendations. For example, the guidelines differ in age at JIA onset cut‐offs, ILAR subtypes, and screening frequency recommendations.^13^ In addition, retrospective studies report contradicting evidence to support each of these clinical risk factors.^5^ A recently published risk prediction model derived from clinical risk factors reported that risk factors with the greatest prediction power were age at JIA onset, ILAR category, and ANA positivity.^13^ There is an unmet need to include novel biomarkers and/or incorporate genotyping to improve screening for JIAU.^10^

Both JIA and JIAU are multifactorial autoimmune diseases with contributions from genetic and environmental risk factors.^14^, ^15^ Associations with HLA genes are considered biomarkers of uveitis.^16^ The most widely reported HLA association in JIAU is *HLA‐DRB1*, recently fine‐mapped to the amino acid position 11. Interestingly, the highly correlated amino acids at positions 11 and 13 of HLA‐DRB1 are the major genetic risk factor for susceptibility to RF‐negative polyarticular and oligoarticular JIA.^17^ These positions at HLA‐DRB1 form part of the YST motif, an antigen binding groove. Tyrosine at position 10, serine at 11, and threonine at 12 of HLA‐DRB1 make up the amino acids in the YST motif; all three residues within the motif are highly correlated with DRB1 position 13.^3^

Here, we present the largest and most detailed genetic study to date of the HLA region in JIAU. The aims of this study were to define genetic risk of JIAU and to investigate for the first time the relationship of genetic risk factors with known clinical risk factors. The ultimate ambition of this research is to create an integrated approach to refine the prediction of JIAU.

## PATIENTS AND METHODS

### Study cohort

Patients were recruited from the following UK JIA cohort collections: the UK JIA Biologics Register, which includes the BSPAR Etanercept cohort study (BSPAR‐ETN) and the Biologics for Children with Rheumatic Diseases study (BCRD)^18^; Childhood Arthritis Prospective Study (CAPS)^19^; Childhood Arthritis Response to Medication Study (CHARMS)^20^; and the United Kingdom Juvenile Idiopathic Arthritis Genetics Consortium (UKJIAGC).^21^ Participants with JIA were recruited with ethical approval and provided informed consent, including from the Northwest Multi‐centre for Research Ethics Committee (MREC:02/8/104 and MREC:99/8/84), West Midlands Multi‐Centre Research Ethics Committee (MREC:02/7/106), North‐West Research Ethics Committee (REC:09/H1008/137) and the NHS Research Ethics Committee (REC:05/Q0508/95). For a list of study collaborators, please see Appendix A.

### Genotyping and imputation

A total of 3,076 JIA samples (579 patients with JIAU and 2,497 patients with JIA without uveitis) were included in this study and genotyped using the Illumina Infinium CoreExome array as described previously (Table 1).^22^ Uveitis was diagnosed by an ophthalmologist through use of regular screening in all patients with JIA and recorded as yes/no for uveitis. Samples and single nucleotide polymorphisms (SNPs) were subject to stringent quality control (QC) measures. Samples with a call rate <0.98, that had discrepancy between genetically inferred sex and database records, that had related individuals in the cohort detected by identity‐by‐descent (IBD), and that had ancestral outliers of non‐European ancestry identified by principal component analysis (PCA) were excluded from the cohort. For related individuals identified through IBD, the individual with the highest call rate was retained for the study. PCA was performed using the flashpca software package (version 2.0), for which outliers were identified using aberrant R library (version 1.0).^23^, ^24^ Sample numbers excluded at each QC step are summarized in Supplementary Figure 1. SNPs were excluded if they were nonautosomal, had a call rate <0.98, and had a minor allele frequency (MAF) <0.01. The dataset was restricted to high‐quality SNP genotypes within the major histocompatibility complex (MHC) on chromosome 6 (29–34 Mb hg build 19). Two‐ and four‐digit HLA alleles, amino acid residues, and SNPs were imputed using the SNP2HLA software package (version 1.0.3).^25^ SNP2HLA uses Beagle for phasing and imputation using the T1DG reference panel. The imputed dataset was filtered for variants with an information score >0.9 and MAF >1%.^25^

**Table 1:** Number and percentage of individuals in the cohort with JIA without uveitis and JIAU by sex, ANA status, and ILAR subtype*

Characteristics	JIA without uveitis, n (%)	JIAU, n (%)
Total	2,497 (81)	579 (19)
Sex		
Female	1,646 (66)	414 (72)
Male	850 (34)	165 (28)
Missing	1 (0)	0 (0)
ANA status		
Positive	500 (20)	196 (34)
Negative	529 (21)	61 (11)
Missing	1,468 (59)	322 (56)
ILAR		
Systemic JIA	188 (8)	8 (1)
Persistent oligoarthritis	627 (25)	195 (34)
Extended oligoarthritis	317 (13)	152 (26)
RF‐negative polyarthritis	650 (26)	132 (23)
RF‐positive polyarthritis	151 (6)	15 (3)
ERA	187 (7)	27 (5)
PsA	192 (8)	26 (4)
Undifferentiated JIA	114 (5)	16 (3)
Missing	71 (3)	8 (1)

* The total number of individuals in the cohort was 3,076. ANA, antinuclear antibody; ERA, enthesis‐related arthritis; JIA, juvenile idiopathic arthritis; JIAU, JIA‐associated uveitis; ILAR, International League of Associations for Rheumatology; PsA, juvenile psoriatic arthritis; RF, rheumatoid factor.

### Genetic association testing

Association testing was performed with logistic regression assuming an additive model of imputed allele dosages, in which reported odds ratios reflect an additive effect per allele carried. The omnibus test was used for association testing at multiallelic markers, for which the most frequent allele was selected as the reference. To account for residual population substructure, three principal components were included in all analyses. A study‐wide significance threshold was defined using a Bonferroni‐corrected Type I error rate based on the number of markers in the final dataset. To detect independent effects from initial association signals, forward stepwise logistic regression and conditional analysis were used. These analyses were then repeated in a subset of the dataset consisting only of the persistent and extended oligoarthritis ILAR subtypes. To conduct sex dimorphism analysis, an association test using sex as an interaction term was used.^3^ Deviation from the Hardy‐Weinberg equilibrium (HWE) was tested for each reported genetic variant in available data of 9,196 population controls in a previously described study.^22^

### Statistical analysis of risk factors

In a subset of the cohort with complete data, we tested the association of clinical risk factors prespecified from the literature for JIAU, including age of JIA onset, sex, ILAR subtype, and ANA status in a univariate analysis using logistic regression.^13^, ^26^ Sample numbers for these clinical risk factors can be seen in Supplementary Table 1. A multivariable analysis was performed to create a fully adjusted model accounting for correlation among the risk factors. Genetic risk factors were added to the multivariable model to test for independence, and the improvement in model fit was assessed using a likelihood ratio test (LRT). We derived a final main effects model by performing forwards stepwise regression with 10‐fold cross‐validation where the final model was defined by minimizing Akaike information criterion (AIC). Classification performance of this model was estimated using area under the receiver operator characteristic curve (AUC); this was compared with the AUC derived from a model based solely on clinical risk factors. Differences in effect estimates for the genetic and clinical variables used in this analysis between the samples retained in the complete case analysis (complete case group) and those excluded for missing data (incomplete case group) were tested by including a variable for group membership as an interaction term. A statistically significant interaction term was interpreted as evidence for difference in the effect sizes between the groups. All analyses were performed using R version 3.6.

## RESULTS

### Three independent associations within HLA genes

The post‐QC dataset consisted of 3,076 patients with JIA, including 579 with uveitis and 2,479 without uveitis with a total of 7,773 high‐quality imputed variants. The Bonferroni study‐wide significance threshold was defined as 6.43 × 10^−6^, and an odds ratio (OR) >1 is considered a risk for JIAU.

**Figure 1:**
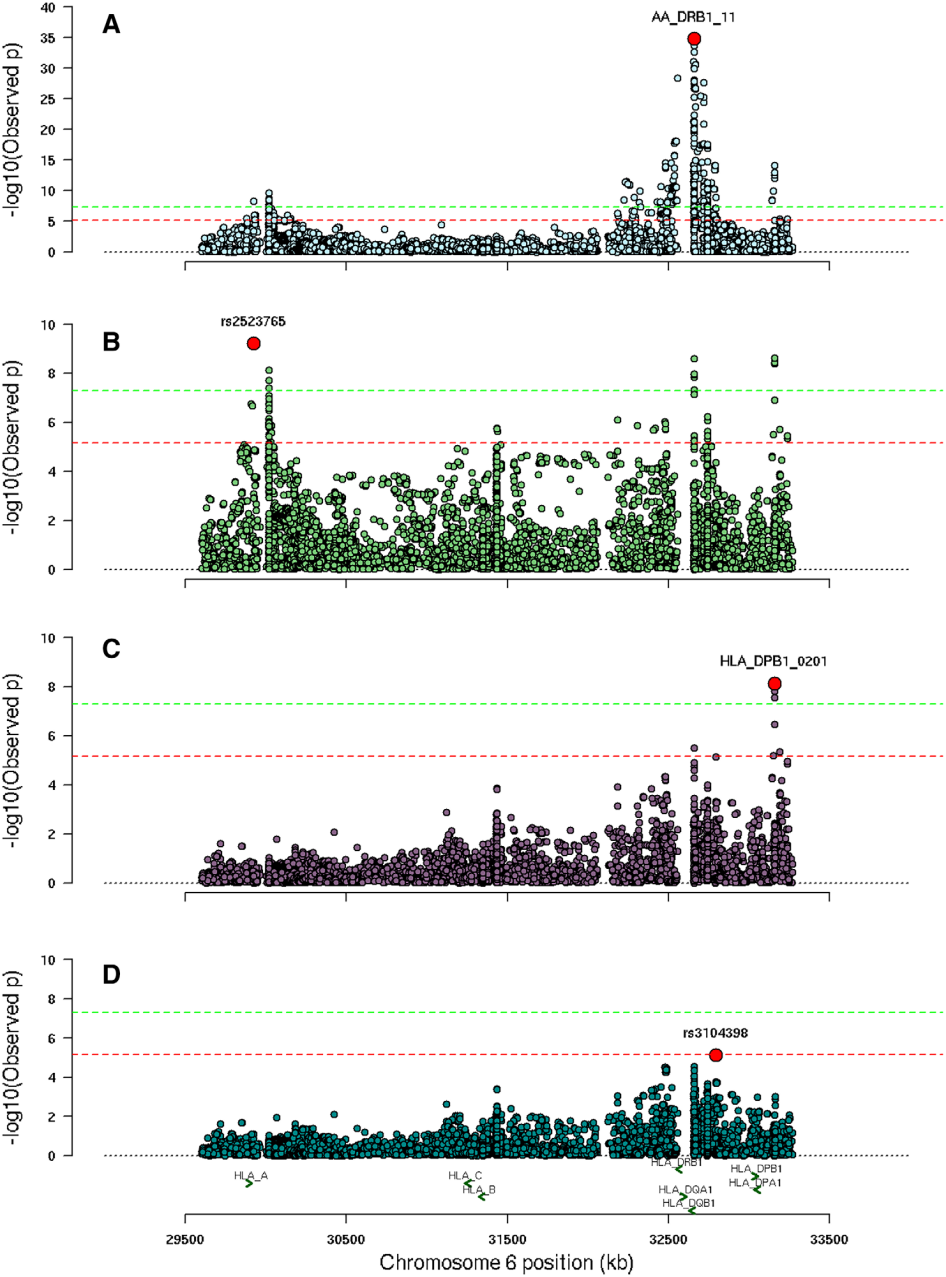
Manhattan plots for independent associations within the HLA region. (A) Position 11 of HLA‐DRB1 is lead marker for HLA association analysis. (B) *HLA‐A SNP* rs2523765 is lead marker for conditional analysis of position 11 of HLA‐DRB1. (C) HLA‐DPB1*0201 is lead marker for conditional analysis of position 11 of DRB1 and rs2523765. (D) Conditional analysis of three leading HLA markers finds no further study‐wide significant markers. The log10 of the *P* value of each HLA marker (vertical axis) is plotted against the base position of each HLA marker on chromosome 6. SNP, single nucleotide polymorphism.

Fine‐mapping of the HLA region identified three independent genetic risk factors for uveitis susceptibility within JIA. Association testing of all genetic markers in the cohort identified amino acid position 11 of HLA‐DRB1 (*P* = 1.6 × 10^−35^) as the most significantly associated marker (Figure 1A), where serine was associated with increased risk of uveitis (OR 2.2, 95% confidence interval [95%] CI 1.9–2.5). Univariate *P* values and frequencies for each residue at position 11 of HLA‐DRB1 are reported in Supplementary Table 2. This association correlated strongly with that of HLA‐DRB1 position 13 (*P* = 2.2 × 10^−34^), where the amino acids serine (OR 1.6, 95% CI 1.4–1.9) and glycine (OR 1.9, 95% CI 1.5–2.3) conferred the most risk of uveitis (Supplementary Table 3). The most associated classical HLA allele was HLA‐DPB1*0201 (*P* = 7.3 × 10^−15^). HLA‐B*27 has previously been reported as a risk factor for anterior uveitis in JIA^15^; however, it was not significantly associated in this JIAU cohort (*P* = 0.85).

Conditional analysis on position 11 of HLA‐DRB1 revealed an independent signal at the SNP rs2523765 close to *HLA‐A* (*P* = 5.8 × 10^−10^, OR 0.6, 95% CI 0.5–0.7) (Figure 1B and Supplementary Table 2). The top amino acid association with HLA‐A is at position 127, where lysine conferred the most risk (*P* = 7.6 × 10^−9^, OR = 1.5, 95% CI 1.3–1.7). Conditioning on HLA‐DRB1 position 11 and rs2523765 revealed an independent signal at HLA‐DPB1*0201 (*P* = 7.2 × 10^−9^, OR 1.6, 95% CI 1.4–1.9) (Figure 1C and Supplementary Table 2). The top amino acid association at this gene is with position 69 (*P* = 2.8 × 10^−8^). Further conditional analysis revealed the signal at rs3104398 (*P* = 7.2 × 10^−6^), which was below the threshold for study‐wide significance (Figure 1D). Table 2 summarizes the associations and effect estimates of residues at position 11 of HLA‐DRB1, rs2523765, and HLA‐DPB1*0201 from a fully adjusted multivariable model. None of the reported genetic variants deviated from the HWE (*P* value >0.05; Supplementary Table 4), and there was no significant difference in effect estimates between the complete case group and incomplete case group (Supplementary Table 5).

**Table 2:** Associations and effect estimates for each independent association from a fully adjusted multivariable model including HLA‐DRB1 position 11 residues, rs2523765 (*HLA‐A)*, and *HLA‐DPB1* * *0201* *

Gene	Amino acid position/BP	Residue/allele	Frequency of JIAU	Frequency of JIA without uveitis	*P* value	OR	95% CI
		proline	0.11	0.15	7.04 × 10^−3^	0.75	0.60–0.92
HLA‐DRB1	11 (ref serine)	valine	0.05	0.15	7.73 × 10^−18^	0.31	0.24–0.40
		glycine	0.04	0.09	7.78 × 10^−8^	0.40	0.28–0.53
		leucine	0.09	0.16	2.86 × 10^−11^	0.46	0.37–0.58
		aspartate	0.02	0.02	5.51 × 10^−1^	0.87	0.54–1.35
HLA‐A (rs2523765)	29925085	‐	0.30	0.39	3.13 × 10^−9^	0.64	0.55–0.74
HLA‐DPB1	‐	0201	0.26	0.16	3.04 × 10^−9^	1.67	1.41–1.98

* The effect estimates are adjusted for all other selected variants. OR >1 implies increased risk of JIAU. Serine is the reference for residues at HLA‐DRB1 position 11 therefore ORs are relative to serine. BP, base position; JIA, juvenile idiopathic arthritis; JIAU, JIA‐associated uveitis; OR; odds ratio; 95% CI, 95% confidence interval.

### Genetic factors refine risk in JIA ILAR subgroups

JIA ILAR subtype is a clinical risk factor for JIAU; specifically, BSPAR screening guidelines consider individuals with oligoarthritis to be at high risk for JIAU onset.^9^ Therefore, we explored the potential of genetic associations to refine the risk of JIAU onset in the ILAR subtype oligoarthritis. When restricted to individuals with oligoarthritis, the cohort included 1,291 individuals; 347 had JIAU, and 944 had JIA without uveitis. Association testing on all *HLA* alleles in a combined group of persistent and extended oligoarthritis JIA ILAR subtypes revealed that position 11 of HLA‐DRB1 was again the strongest signal, but with a more modest significance (*P* = 5.1 × 10^−14^). Serine at position 11 suggested the most risk of uveitis onset (OR 1.8, 95% CI 1.5–2.2). Conditioning on position 11 of HLA‐DRB1 in the oligoarticular cohort revealed no further study‐wide significant signals. These results suggest that genetic risk factors can further refine risk of uveitis in individuals considered to be at high risk for JIAU because of ILAR classification.

### No evidence for sex dimorphism at HLA‐DRB1 position 11

The genetic risk marker at position 11 of HLA‐DRB1 has previously been reported to be a female‐specific effect for JIAU, and this study aimed to validate this finding in this cohort.^3^ The cohort was restricted to a dataset with girls and young women (with JIAU 414, with JIA without uveitis 1,646) and a dataset with boys and young men (with JIAU 165, with JIA without uveitis 850). We found the HLA‐DRB1 position 11 to be significantly associated with JIAU in both a restricted dataset of female samples (*P* = 3.5 × 10^−20^) and a restricted dataset of male samples (*P* = 4.4 × 10^−8^) with similar effect estimates of position 11 residues in both sexes (Supplementary Figure 2). An association test for serine at position 11 with sex as an interaction term was implemented on the total cohort, which revealed no evidence that this signal was sex dimorphic in the cohort used in this study (*P* = 0.16).

### Genetic risk factors improve risk classification of uveitis in combination with clinical risk factors

A univariate analysis was conducted for each of the prespecified clinical risk factors: AAO, ANA status, sex, and ILAR classification. Male was used as the reference for sex, and oligoarthritis was used as the reference for ILAR subtype. All four clinical risk factors were associated with uveitis; the top associated clinical risk factor was AAO (*P* = 9.7 × 10^−38^, OR 0.84), which suggests that an older age at the onset of JIA is associated with decreased risk of JIAU (Supplementary Table 6). A multivariable model of the four clinical risk factors, in a complete case analysis of 217 patients with JIA with uveitis and 874 patients with JIA without uveitis, revealed that sex was no longer significantly associated (*P* = 0.25) with uveitis when adjusting the effect of the other risk factors (Table 3). There was no significant difference in effect estimates for these clinical variables between the complete case group and the incomplete case group (Supplementary Table 7). The three genetic risk factors remain associated when added to the multivariable model that included the four clinical risk factors, which demonstrates that the genetic markers are independent (Table 4). Forward and backward stepwise regression both identified the same best‐fitting model, which included AAO, ANA status, ILAR category, and the three genetic risk factors as selected by AIC. Risk classification of uveitis using 10‐fold cross‐validation on the four prespecified clinical factors was found to have an AUC of 0.71 (95% CI 0.68–0.76). The addition of the three genetic risk factors improved classification performance with an AUC of 0.75 (95% CI 0.71–0.79) and significantly improves the overall fit of the statistical model for uveitis (LRT *P* value = 3.3 × 10^−8^).

**Table 3:** Associations and effect estimates from multivariate analysis of the clinical risk factors sex, ANA status, AAO, and ILAR subtype*

Risk factor	*P* value	OR	95% CI
Sex (ref males)	2.57 × 10^−1^	0.81	0.56–1.17
ANA status	2.74 × 10^−8^	2.79	1.96–4.04
AAO	2.69 × 10^−8^	0.87	0.83–0.91
ILAR (ref oligoarthritis)			
Systemic JIA	3.80 × 10^−3^	0.17	0.04–0.48
RF‐negative polyarthritis	1.24 × 10^−2^	0.62	0.43–0.90
RF‐positive polyarthritis	4.00 × 10^−2^	0.32	0.09–0.85
ERA	7.87 × 10^−1^	0.88	0.34–2.03
PsA	6.84 × 10^−1^	1.15	0.57–2.19
Undifferentiated JIA	8.30 × 10^−1^	1.11	0.41–2.67

* Male was used as the reference for sex, and oligoarthritis was used as the reference for ILAR subtype. OR >1 implies increased risk of JIAU. AAO, age at onset; ANA, antinuclear antibody; ERA, enthesitis‐related arthritis; ILAR, International League of Associations for Rheumatology; JIA, juvenile idiopathic arthritis; JIAU, JIA‐associated uveitis; OR, odds ratio; PsA, juvenile psoriatic arthritis; RF; rheumatoid factor; 95% CI, 95% confidence interval.

**Table 4:** Associations and effect estimates from multivariate analysis of the clinical risk factors sex, ANA status, AAO, and ILAR subtype and the genetic markers HLA‐DRB1 position 11 amino acid residues, *HLA‐DPB1* * *0201*, and rs2523765 *(HLA‐A)* *

Risk factor/genetic marker	*P* value	OR	95% CI
Sex (ref males)	1.47 × 10^−1^	0.75	0.51–1.11
ANA status	3.12 × 10^−5^	2.23	1.54–3.26
AAO	4.18 × 10^−7^	0.88	0.84–0.92
ILAR (ref oligoarthritis)			
Systemic JIA	3.80 × 10^−3^	0.17	0.04–0.48
RF‐negative polyarthritis	1.24 × 10^−2^	0.62	0.43–0.90
RF‐positive polyarthritis	4.00 × 10^−2^	0.32	0.09–0.85
ERA	7.87 × 10^−1^	0.88	0.34–2.03
Psoriatic arthritis	6.84 × 10^−1^	1.15	0.57–2.19
Undifferentiated JIA	8.30 × 10^−1^	1.11	0.41–2.67
HLA‐DRB1 position 11 proline	6.77 × 10^−1^	0.92	0.62–1.35
HLA‐DRB1 position 11 valine	5.25 × 10^−3^	0.51	0.31–0.80
HLA‐DRB1 position 11 glycine	1.14 × 10^−1^	0.62	0.34–1.09
HLA‐DRB1 position 11 leucine	8.86 × 10^−5^	0.42	0.27–0.64
HLA‐DRB1 position 11 aspartate	6.50 × 10^−1^	0.84	0.38–1.74
HLA DPB1*0201	1.84 × 10^−2^	1.42	1.06–1.90
*HLA‐A rs2523765*	2.22 × 10^−4^	0.61	0.46–0.79

* OR >1 implies increased risk of JIAU. Male was used as the reference for sex, oligoarthritis was used as the reference for ILAR subtype, and serine was used as the reference for position 11 of HLA‐DRB1. AAO, age at onset; ANA, antinuclear antibody; ERA, enthesitis‐related arthritis; ILAR, International League of Associations for Rheumatology; JIA, juvenile idiopathic arthritis; JIAU, JIA‐associated uveitis; OR, odds ratio; RF, rheumatoid factor; 95% CI, 95% confidence interval.

## DISCUSSION

This study, using a large dataset of patients with JIA with and without uveitis, identified three independent genetic risk factors for JIAU. These were shown to be independent of the well‐established clinical risk factors and were found to improve the classification of uveitis in a model combining clinical and genetic risk factors. This study provides evidence to support further research to potentially adapt screening tools to include newly discovered biomarkers as recommended by expert review.^10^

This research will help further define the genetic risk of uveitis in patients with JIA. HLA‐DRB1 at positions 11 and 13 have been associated with JIAU previously; this study has independently validated these associations. Haasnoot et al^3^ reported that the presence of serine at position 11 of HLA‐DRB1 is correlated with JIAU, and Heiligenhaus et al^27^ reported that the position 11 of HLA‐DRB1 is a good predictor of uveitis in patients with JIA. We also report that serine at position 11 of HLA‐DRB1 is correlated with uveitis in patients with JIA. A second association signal at position 13 of HLA‐DRB1 was detected in the Haasnoot et al study,^3^ in which serine or glycine at this position were in linkage disequilibrium with serine at position 11. This finding is consistent in this study; serine or glycine at position 13 were associated with increased risk of uveitis in a cohort of patients with JIA and JIAU. Forward stepwise logistic regression from HLA‐DRB1 position 11 detected an independent signal at rs2523765 within *HLA‐A* in this cohort. To our knowledge, this independent signal has not been associated with uveitis onset in previous studies. A second independent signal at HLA‐DPB1*0201 was associated with increased risk of JIAU in this cohort. HLA‐DPB1*0201 has been associated with a 7.7‐fold increased risk of chronic uveitis when in combination with HLA‐DRB1*1104.^5^ Interestingly, HLA‐DPB1*0201 has been associated with JIA.^17^, ^28^ The shared association of *HLA* markers in JIA and JIAU onset suggest a pleiotropic effect of genetic risk factors. In addition to the three independent effects, we observe evidence for a significant association with the SNP rs3104398 within the MHC class II region. Although this did not pass our study‐wide significance, it does suggest the possibility of further genetic risk factors for JIAU within this region that could be resolved with larger sample sizes.

These analyses demonstrate that the addition of genetic risk factors can further define the risk of uveitis in patients with JIA. The association with HLA‐DRB1 position 11 was correlated in the oligoarthritis subset of the cohort with an OR of 1.8 compared with 2.2 in all JIA. This demonstrates that the magnitude of effect for serine at position 11 of HLA‐DRB1 is consistent between all subtypes of JIA and oligoarthritis and suggests that individuals with this ILAR subtype may carry genetic risk factors that place them at a higher risk for uveitis. Many studies corroborate that individuals with oligoarthritis ILAR subtypes are high risk for uveitis.^29^ Multivariate analysis of the clinical risk factors AAO, ANA status, sex, and ILAR classification found that AAO and ANA status are strongly associated with JIAU. Additionally, incorporation of the genetic risk factors serine at position 11 of HLA‐DRB1, rs2523765, and HLA‐DPB1*0201 to this model found that genetic risk factors significantly improve the fit of the statistical model for uveitis.

Sex and ILAR subtype are established risk factors for JIAU. This study explored these clinical risk factors alongside genetic risk factors for JIAU. Individual analysis of female patients in the study by Haasnoot et al^3^ found that the association with position 11 of HLA‐DRB1 was a female‐driven signal. The theory that girls and young women are more susceptible to JIAU than boys and young men is supported by a separate study, in which girls and young women were at significantly higher risk of JIAU than boys and young men, particularly if they had an early AAO of arthritis and ANA positivity.^30^ Oligoarticular JIA is often stated as a risk factor for uveitis along with the presence of the risk factor HLA‐DRB1 position 11.^1^ In the study by Haasnoot et al,^3^ the strength of the association signal at position 11 of HLA‐DRB1 did not change from the female‐driven association signal when the analysis was repeated using only oligoarthritis and RF‐negative polyarthritis subtypes. When repeating this sex dimorphism analysis in the cohort in this study, the signal at serine at position 11 of DRB1 was found to not be significantly sex dimorphic for JIAU.

Although the results of this study have identified genetic risk factors for JIAU, it is important to recognize the limitations. One limitation of this study is the lack of representation of non‐European ancestral samples from the dataset. PCA was used during QC of this dataset to exclude ancestral outliers in order to protect against population stratification (Supplementary Figure 1). Because of this QC measure and the relatively small number of non‐European samples in this dataset, non‐European ancestry samples were not included in the analysis. The results of this study are therefore not representative of genetic risk factors for uveitis in non‐European populations, and future research on the genetic risk of JIAU in non‐European populations is required.

An important limitation of this study is the availability of clinical data in all of the datasets and cohorts. As can be seen in Table 1, there are significant missing data for several clinical variables, in particular ANA status. The impact of this is a reduction in the sample size for the integrated model component of this research as a complete case analysis was conducted. Moreover, it is important to highlight that duration of JIA at uveitis diagnosis was not available for all patients and therefore not included in this study. Individuals with a shorter duration of JIA at data collection that were included in this study could go on to develop uveitis. This could potentially result in an underestimation of uveitis cases in this cohort. The authors of this study would also like to acknowledge that eight individuals in this study had cases of systemic JIA with uveitis. Patients with systemic JIA are usually at low risk for JIAU, and consequently, we cannot be certain that the correct ILAR subtype has been assigned in these cases.^31^ However, it was important to include these samples in this study to best stratify genetic risk of uveitis in all patients with JIA.

The results of this study have evidenced the role of genetic risk factors in JIAU and highlight their potential use in defining risk of uveitis in patients with JIA. The incorporation of genetic risk factors into current JIAU screening guidelines could aid prioritization of children and young people at high risk for uveitis, which could facilitate the prevention of severe disease that may lead to permanent visual impairment. However, future research is necessary to validate these findings in a prospective cohort to assess the clinical utility of using genetic risk factors in screening guidelines and future endeavors require the development of a genetic test that can be practically used in the clinic.

## AUTHOR CONTRIBUTIONS

All authors were involved in drafting the article or revising it critically for important intellectual content, and all authors approved the final version to be published. Dr Bowes had full access to all of the data in the study and takes responsibility for the integrity of the data and the accuracy of the data analysis.

### Study conception and design

Tordoff, Smith, Lawson‐Tovey, Dick, Beresford, Ramanan, Hyrich, Morris, Eyre, Wedderburn, Bowes.

### Acquisition of data

Tordoff, Smith, Lawson‐Tovey, Dick, Beresford, Ramanan, Hyrich, Morris, Eyre, Wedderburn, Bowes.

### Analysis and interpretation of data

Tordoff, Bowes.

## Supporting information


Disclosure form

**Supplementary table 1:** Number and percentage number of complete cases versus incomplete cases in the cohort. Incomplete cases represent individuals with missing data for sex, ANA status, age at onset (AAO) or ILAR classification and were not included in the combined genetic and clinical risk factor analysis in this manuscript. Total number of complete cases = 1091, total number of incomplete cases = 1985. JIA; juvenile idiopathic arthritis, JIAU; JIA associated uveitis, RF; rheumatoid factor, ERA; enthesitis related arthritis.
**Supplementary Table 2:** associations and effect estimates for each independent association. Univariate results are presented for each residue at HLA‐DRB1 amino acid position 11, results for HLA‐A (rs2523765) are presented conditioned of all residues at DRB1 position 11 and results for HLA‐DPB1 *0201 are presented conditioned of DRB1 position 11 and HLA‐A. OR >1 implies increased risk to JIAU. Frq.; frequency, OR; odds ratio, CI; confidence interval, BP; base position.
**Supplementary Table 3:** associations and effect estimates for independent associations of residues at position 13 of HLA‐DRB1. Univariate analysis results are presented in JIAU samples and JIA without uveitis samples. Frq.; frequency, OR; odds ratio, CI; confidence interval, BP; base position.
**Supplementary Table 4**: Hardy Weinberg equilibrium (HWE) for healthy controls in the JIA GWAS per genetic risk marker included in the joint genetic and clinical risk factor multivariable model in this study.
**Supplementary Table 5:** Effect estimates for amino acids at position 11 of HLA‐DRB1, HLA‐DPB1*0201 and rs25237665 (HLA‐A) in complete cases versus incomplete cases in the cohort. Interaction P value was calculated for each marker using complete/incomplete as an interaction term. Incomplete cases represent individuals with missing data for sex, ANA status, age at onset (AAO) or ILAR classification and were not included in the combined genetic and clinical risk factor analysis in this manuscript Serine was used as the reference for the multivariable model of genetic risk factors presented in this table. Total number of complete cases = 1091, total number of incomplete cases = 1985.
**Supplementary Table 6:** associations and effect estimates from univariate analysis of the clinical risk factors sex, ANA status, AAO and ILAR subtype. Male was used as the reference for sex and oligoarthritis was used as the reference for ILAR subtype. AAO; age at onset, OR; odds ratio, RF; rheumatoid factor, ERA; enthesitis related arthritis.
**Supplementary Table 7:** associations and effect estimates from univariate analysis of the clinical risk factors sex, ANA status, AAO and ILAR subtype in complete and incomplete subsets of the data. Male was used as the reference for sex and oligoarthritis was used as the reference for ILAR subtype. Interaction P value was calculated for each risk factor in the whole dataset using complete/incomplete as an interaction term. Incomplete cases represent individuals with missing data for sex, ANA status, AAO or ILAR classification and were not included in the combined genetic and clinical risk factor analysis in this manuscript. Total number of complete cases = 1091, total number of incomplete cases = 1985.OR; odds ratio, RF; rheumatoid factor, ERA; enthesitis related arthritis.
**Supplementary Figure 1:** quality control (QC) of individual level genotype data illustrating the exclusions made at each sample‐level QC threshold. Exclude 1‐ Total of 355 individuals excluded due to low genotype call rate (<98%) and heterozygosity (3 SD). Exclude 2‐ Total of 39 individuals excluded due to discrepancy between genetically inferred sex and recorded sex. Exclude 3‐ Total of 287 individuals excluded by IBD indicating high levels of relatedness. Exclude 4‐ Total of 152 individuals excluded by PCA and the detection of ancestral outliers. JIA: juvenile idiopathic arthritis, SD: standard deviations, PCA: principal component analysis, HM3: HapMap 3. *IBD exclusions reflect participants recruited to multiple studies under different patient nomenclature schemes.
**Supplementary Figure 2:** Effect sizes (ORs and 95% CIs) of each residue at position 11 of *HLA‐DRB1* in male and female cohorts. Ser; serine, Asp; aspartate, Pro; proline, Leu; leucine, Gly; glycine, Val; valine. The effect sizes of each residue in females (vertical axis) is plotted against the effect sizes of each residue in males (horizontal axis). Error bars represent the 95% confidence interval.

## APPENDIX A MEMBERS OF THE CLUSTER CONSORTIUM

Members of the CLUSTER Consortium are as follows: Prof Lucy R. Wedderburn, Ms Zoe Wanstall, Ms Vasiliki Alexiou, Mr Fatjon Dekaj, Ms Bethany R. Jebson, Dr Melissa Kartawinata, Ms Aline Kimonyo, Ms Eileen Hahn, Ms Genevieve Gottschalk, Ms Freya Luling Feilding, Ms Alyssia McNeece, Ms Fatema Merali, Ms Elizabeth Ralph, Ms Emily Robinson, Ms Emma Sumner (UCL GOS Institute of Child Health, London); Prof Andrew Dick (UCL Institute of Ophthalmology, London); Prof Michael W. Beresford, Dr Emil Carlsson, Dr Joanna Fairlie, Dr Jenna F. Gritzfeld, Dr Oliver McClurg, Dr Karen Rafferty (University of Liverpool); Prof Athimalaipet V. Ramanan, Ms Teresa Duerr (University Hospitals Bristol and Weston NHS Foundation Trust); Prof Michael Barnes, Ms Sandra Ng (Queen Mary University, London); Prof Kimme Hyrich, Prof Stephen Eyre, Prof Soumya Raychaudhuri, Prof Wendy Thomson, Dr John Bowes, Ms Jeronee Jennycloss, Ms Saskia Lawson‐Tovey, Dr Paul Martin, Prof Andrew Morris, Dr Stephanie Shoop‐Worrall, Dr Samantha Smith, Mr Michael Stadler, Dr Damian Tarasek, Dr Melissa Tordoff, Dr Annie Yarwood (University of Manchester); Dr Chris Wallace, Dr Wei‐Yu Lin (University of Cambridge); Prof Nophar Geifman (University of Surrey); Dr Sarah Clarke (School of Population Health sciences and MRC Integrative Epidemiology Unit, University of Bristol); Dr Thierry Sornasse (AbbVie Inc.); Dr Robert J. Benschop, Dr Rona Wang (Eli Lilly); Dr Daniela Dastros‐Pitei, Dr Sumanta Mukherjee, (GlaxoSmithKline Research and Development Limited); Dr Michael McLean, Dr Anna Barkaway (Pfizer); Dr Peyman Adjamian (Swedish Orphan Biovitrum AB [publ] [Sobi]); Helen Neale (UCB Biopharma SRL); the CLUSTER Champions.
