# Supplementary material for: Integration of Genetic and Clinical Risk Factors for Risk Classification of Uveitis in Patients With Juvenile Idiopathic Arthritis
Source: Arthritis Rheumatol. 2024 Sep 5;76(12):1789–96. doi: 10.1002/art.42955 (PMC11605271; doi:10.1002/art.42955)
Supplement: Supplementary file 2 — Supplementary table 1: Number and percentage number of complete cases versus incomplete cases in the cohort. Incomplete cases represent individuals with missing data for sex, ANA status, age at onset (AAO) or ILAR classification and were not included in the combined genetic and clinical risk factor analysis in this manuscript. Total number of complete cases = 1091, total number of incomplete cases = 1985. JIA; juvenile idiopathic arthritis, JIAU; JIA associated uveitis, RF; rheumatoid factor, ERA; enthesitis related arthritis. Supplementary Table 2: associations and effect estimates for each independent association. Univariate results are presented for each residue at HLA‐DRB1 amino acid position 11, results for HLA‐A (rs2523765) are presented conditioned of all residues at DRB1 position 11 and results for HLA‐DPB1 *0201 are presented conditioned of DRB1 position 11 and HLA‐A. OR >1 implies increased risk to JIAU. Frq.; frequency, OR; odds ratio, CI; confidence interval, BP; base position. Supplementary Table 3: associations and effect estimates for independent associations of residues at position 13 of HLA‐DRB1. Univariate analysis results are presented in JIAU samples and JIA without uveitis samples. Frq.; frequency, OR; odds ratio, CI; confidence interval, BP; base position. Supplementary Table 4: Hardy Weinberg equilibrium (HWE) for healthy controls in the JIA GWAS per genetic risk marker included in the joint genetic and clinical risk factor multivariable model in this study. Supplementary Table 5: Effect estimates for amino acids at position 11 of HLA‐DRB1, HLA‐DPB1*0201 and rs25237665 (HLA‐A) in complete cases versus incomplete cases in the cohort. Interaction P value was calculated for each marker using complete/incomplete as an interaction term. Incomplete cases represent individuals with missing data for sex, ANA status, age at onset (AAO) or ILAR classification and were not included in the combined genetic and clinical risk factor analysis in this manuscr [file ART-76-1789-s002.docx]

**Integrated Risk Classification of JIA-Uveitis**

**Integration of genetic and clinical risk factors improves the risk classification of uveitis in patients with juvenile idiopathic arthritis.**

**Melissa Tordoff, PhD^1^, Samantha L. Smith, PhD^1^, Saskia Lawson-Tovey, BA^1,2^, UK JIA Biologics Register, CAPS, CHARMS, CLUSTER, JIAGC, Andrew D. Dick, M.D ^4,5,6^, Michael W. Beresford, PhD^7,8,9^, Athimalaipet V. Ramanan FRCP ^4,10^, Kimme L Hyrich M.D, PhD^2,3^, Andrew P Morris, PhD^1,2^, Stephen Eyre, PhD^1,2^, Lucy R Wedderburn MD, PhD, FRCP ^11,12,13^, John Bowes, PhD^1,2^ and on behalf of the CLUSTER consortium. Address correspondence to John Bowes, PhD, Centre for Genetics and Genomics Versus Arthritis, University of Manchester, Manchester, United Kingdom, M139PT. Email: j.bowes@manchester.ac.uk.**

^1^Centre for Genetics and Genomics Versus Arthritis, Centre for Musculoskeletal Research, Manchester Academic Health Science Centre, The University of Manchester, Manchester, UK. ^2^National Institute of Health Research Manchester Biomedical Research Centre, Manchester Academic Health Science Centre, Manchester University NHS Foundation Trust, Manchester, UK. ^3^Centre for Epidemiology Versus Arthritis, The University of Manchester, Manchester, UK. ^4^Translational Health Sciences, University of Bristol, Bristol BS2 8DZ, UK. ^5^UCL Institute of Ophthalmology, London EC1V 9EL, UK. ^6^NIHR Biomedical Research Centre, Moorfields Eye Hospital, London EC1V 2PD, UK. ^7^Department of Women’s and Children’s Health, Institute of Life Course and Medical Sciences, University of Liverpool, Liverpool L14 5AB, UK. ^8^Department of Rheumatology, Alder Hey Children’s NHS Foundation Trust Hospital, Liverpool L14 5AB, UK. ^9^National Institute for Health Research Alder Hey Clinical Research Facility, Alder Hey Children’s NHS Foundation Trust Hospital, Liverpool L14 5AB, UK. ^10^Bristol Royal Hospital for Children, Bristol, UK. ^11^Infection, Immunity and Inflammation Research and Teaching Department, UCL Great Ormond Street Institute of Child Health, London, UK. ^12^Centre for Adolescent Rheumatology Versus Arthritis at UCL, UCL Hospital and Great Ormond Street Hospital, London, UK. ^13^NIHR Biomedical Research Centre at Great Ormond Street Hospital, London, UK.

**Supplementary Material**

|  |  | Complete Cases | Incomplete Cases |
| --- | --- | --- | --- |
| Uveitis | JIA without uveitis | 874 (80.1) | 1623 (81.8) |
|  | JIAU | 217 (19.9) | 362 (18.2) |
| Sex | Female | 738 (67.6) | 1322 (66.6) |
|  | Male | 353 (32.4) | 662 (33.4) |
|  | Missing | 0 (0) | 1 (0.1) |
| ANA Status | Positive | 593 (54.4) | 103 (52.8) |
|  | Negative | 498 (45.6) | 92 (47.2) |
|  | Missing | 0 (0) | 1790 (90.2) |
| AAO | ≤6 | 571 (52.3) | 840 (42.3) |
|  | >6 | 520 (47.7) | 868 (43.7) |
|  | Missing | 0 (0) | 277 (14) |
| ILAR | Systemic | 76 (7) | 120 (6) |
|  | Oligoarthritis | 470 (43.1) | 821 (41.4) |
|  | RF-negative polyarthritis | 335 (30.7) | 447 (22.5) |
|  | RF-positive polyarthritis | 52 (4.8) | 114 (5.7) |
|  | ERA | 59 (5.4) | 155 (7.8) |
|  | Psoriatic arthritis | 70 (6.4) | 148 (7.5) |
|  | Undifferentiated | 29 (2.7) | 101 (5.1) |
|  | Missing | 0 (0) | 79 (4) |
|  | Total | 1091 | 1985 |

**Supplementary table 1:** Number and percentage number of complete cases versus incomplete cases in the cohort. Incomplete cases represent individuals with missing data for sex, ANA status, age at onset (AAO) or ILAR classification and were not included in the combined genetic and clinical risk factor analysis in this manuscript. Total number of complete cases = 1091, total number of incomplete cases = 1985. JIA; juvenile idiopathic arthritis, JIAU; JIA associated uveitis, RF; rheumatoid factor, ERA; enthesitis related arthritis.

| Gene | Amino acid position/BP | Residue/allele | Frq. JIAU | Frq. JIA without uveitis | p-value | OR | 95% CI |
| --- | --- | --- | --- | --- | --- | --- | --- |
|  |  | Serine | 0.67 | 0.48 | 1.61x10-28 | 2.15 | 1,88-2.46 |
|  |  | Proline | 0.11 | 0.11 | 0.95 | 1.01 | 0.82-1.23 |
| HLA-DRB1 | 11 | Valine | 0.05 | 0.15 | 6.82x10-14 | 0.37 | 0.28-0.48 |
|  |  | Glycine | 0.04 | 0.09 | 3.30x10-7 | 0.45 | 0.33-0,60 |
|  |  | Leucine | 0.09 | 0.16 | 4.35x10-8 | 0.54 | 0.43-0.67 |
|  |  | Aspartate | 0.02 | 0.02 | 0.13 | 1.40 | 0.89-2.15 |
| HLA-A (rs2523765) | 29925085 | - | 0.30 | 0.39 | 5.8x10-10 | 0.64 | 0.55-0.74 |
| HLA-DPB1 | - | 0201 | 0.26 | 16 | 7.27x10-09 | 1.67 | 1.41-1.98 |

**Supplementary Table 2:** associations and effect estimates for each independent association. Univariate results are presented for each residue at HLA-DRB1 amino acid position 11, results for HLA-A (rs2523765) are presented conditioned of all residues at DRB1 position 11 and results for *HLA-DPB1 *0201* are presented conditioned of DRB1 position 11 and HLA-A. OR >1 implies increased risk to JIAU. Frq.; frequency, OR; odds ratio, CI; confidence interval, BP; base position.

| Gene | Amino acid position/BP | Residue/allele | Frq. JIAU | Frq. JIA without uveitis | p-value | OR | 95% CI |
| --- | --- | --- | --- | --- | --- | --- | --- |
|  |  | Phenylalanine | 0.17 | 0.14 | 5.83x10-07 | 0.60 | 0.49-0.73 |
|  |  | Glycine | 0.11 | 0.04 | 1.46x10-11 | 1.91 | 1.58-2.31 |
| HLA-DRB1 | 13 | Histidine | 0.13 | 0.19 | 4.87x10-13 | 0.38 | 0.29-0.49 |
|  |  | Arginine | 0.11 | 0.15 | 9.52x10-01 | 1.01 | 0.81-1.23 |
|  |  | Serine | 0.40 | 0.33 | 2.64x10-14 | 1.68 | 1.47-1.92 |
|  |  | Tyrosine | 0.08 | 0.14 | 3.29x10-07 | 0.44 | 0.32-0.60 |

**Supplementary Table 3:** associations and effect estimates for independent associations of residues at position 13 of HLA-DRB1. Univariate analysis results are presented in JIAU samples and JIA without uveitis samples. Frq.; frequency, OR; odds ratio, CI; confidence interval, BP; base position.

| Genetic Marker | HWE healthy controls (p) |
| --- | --- |
| HLA-DRB1 pos 11 Serine | 0.93 |
| HLA-DRB1 pos 11 Proline | 0.81 |
| HLA-DRB1 pos 11 Valine | 0.60 |
| HLA-DRB1 pos 11 Glycine | 0.55 |
| HLA-DRB1 pos 11 Leucine | 0.96 |
| HLA-DRB1 pos 11 Aspartate | 0.67 |
| HLA-DPB1*0201 | 0.64 |
| rs2523765 (HLA-A) | 0.17 |

**Supplementary Table 4**: Hardy Weinberg equilibrium (HWE) for healthy controls in the JIA GWAS per genetic risk marker included in the joint genetic and clinical risk factor multivariable model in this study.

|  | Complete Cases | | | | Incomplete Cases | | | |  |
| --- | --- | --- | --- | --- | --- | --- | --- | --- | --- |
| Genetic Marker | MAF | OR | CI (95%) | p-value | MAF | OR | CI (95%) | p-value | Interaction p-value |
| HLA-DRB1 pos 11 Serine | 0.46 | - | - | - | 0.50 | - | - | - | 6.5x10-01 |
| HLA-DRB1 pos 11 Proline | 0.12 | 0.71 | 0.49-1.00 | 5.9x10-02 | 0.11 | 0.77 | 0.59-1.00 | 5.8x10-02 | 2.6x10-01 |
| HLA-DRB1 pos 11 Valine | 0.12 | 0.35 | 0.22-0.53 | 2.2x10-06 | 0.14 | 0.29 | 0.21-0.40 | 8.9x10-13 | 3.9x10-01 |
| HLA-DRB1 pos 11 Glycine | 0.07 | 0.45 | 0.25-0.75 | 4.1x10-03 | 0.09 | 0.37 | 0.25-0.54 | 6.0x10-07 | 3.6x10-01 |
| HLA-DRB1 pos 11 Leucine | 0.14 | 0.39 | 0.26-0.58 | 5.5x10-06 | 0.15 | 0.51 | 0.38-0.66 | 1.1x10-06 | 3.3x10-01 |
| HLA-DRB1 pos 11 Aspartate | 0.02 | 0.92 | 0.43-1.84 | 8.1x10-01 | 0.02 | 0.86 | 0.46-1.51 | 6.1x10-01 | 2.7x10-01 |
| HLA-DPB1*0201 | 0.20 | 1.59 | 1.21-2.09 | 8.2x10-04 | 0.17 | 1.74 | 1.39-2.16 | 7.0x10-07 | 4.9x10-01 |
| rs2523765 (HLA-A) | 0.37 | 0.60 | 0.46-0.76 | 4.8x10-05 | 0.37 | 0.66 | 0.55-0.80 | 1.9x10-05 | 2.8x10-01 |

**Supplementary Table 5:** Effect estimates for amino acids at position 11 of HLA-DRB1, HLA-DPB1*0201 and rs25237665 (HLA-A) in complete cases versus incomplete cases in the cohort. Interaction P value was calculated for each marker using complete/incomplete as an interaction term. Incomplete cases represent individuals with missing data for sex, ANA status, age at onset (AAO) or ILAR classification and were not included in the combined genetic and clinical risk factor analysis in this manuscript Serine was used as the reference for the multivariable model of genetic risk factors presented in this table. Total number of complete cases = 1091, total number of incomplete cases = 1985.

| Clinical Risk Factor | p-value | OR | 95% CI |
| --- | --- | --- | --- |
| Sex (ref males) | 1.05x10-02 | 1.30 | 1.06-1.58 |
| ANA status | 1.59x10-14 | 3.40 | 2.50-4.68 |
| Age at onset | 9.77x10-38 | 0.84 | 0.81-0.86 |
| ILAR (ref oligoarthritis) | - | - | - |
| Systemic | 3.96x10-09 | 0.12 | 0.05-0.22 |
| RF-negative polyarthritis | 2.07x10-07 | 0.55 | 0.44-0.69 |
| RF-positive polyarthritis | 2.50x10-06 | 0.27 | 0.15-0.45 |
| ERA | 1.41x10-05 | 0.39 | 0.25-0.58 |
| Psoriatic arthritis | 4.73x10-06 | 0.37 | 0.23-0.55 |
| Undifferentiated | 4.47x10-04 | 0.38 | 0.21-0.63 |

**Supplementary Table 6****:** associations and effect estimates from univariate analysis of the clinical risk factors sex, ANA status, AAO and ILAR subtype. Male was used as the reference for sex and oligoarthritis was used as the reference for ILAR subtype. AAO; age at onset ,OR; odds ratio, RF; rheumatoid factor, ERA; enthesitis related arthritis.

|  | Complete Cases | | Incomplete Cases | | Interaction of complete | | |
| --- | --- | --- | --- | --- | --- | --- | --- |
| Clinical Risk Factor | OR | CI (95%) | OR | CI (95%) | OR | CI (95%) | p-value |
| Sex (ref males) | 1.18 | 0.86-1.64 | 1.37 | 1.07-1.76 | 0.90 | 0.75-1.09 | 0.28 |
| ANA status | 3.51 | 2.51-4.99 | 2.89 | 1.38-6.42 | 1.06 | 0.71-1.55 | 0.77 |
| Age at onset | 0.86 | 0.82-0.90 | 0.83 | 0.80-0.86 | 0.94 | 0.77-1.15 | 0.54 |
| ILAR (ref oligoarthritis) | - | - | - | - | 0.94 | 0.77-1.13 | 0.49 |
| Systemic | 0.12 | 0.03-0.32 | 0.12 | 0.04-0.26 | - | - | - |
| RF-negative polyarthritis | 0.58 | 0.41-0.83 | 0.53 | 0.40-0.71 | - | - | - |
| RF-positive polyarthritis | 0.23 | 0.07-0.59 | 0.29 | 0.14-0.52 | - | - | - |
| ERA | 0.38 | 0.15-0.80 | 0.40 | 0.24-0.64 | - | - | - |
| Psoriatic arthritis | 0.70 | 0.36-1.26 | 0.24 | 0.12-0.42 | - | - | - |
| Undifferentiated | 0.89 | 0.34-2.03 | 0.26 | 0.12-0.50 | - | - | - |

**Supplementary Table 7:** associations and effect estimates from univariate analysis of the clinical risk factors sex, ANA status, AAO and ILAR subtype in complete and incomplete subsets of the data. Male was used as the reference for sex and oligoarthritis was used as the reference for ILAR subtype. . Interaction P value was calculated for each risk factor in the whole dataset using complete/incomplete as an interaction term. Incomplete cases represent individuals with missing data for sex, ANA status, AAO or ILAR classification and were not included in the combined genetic and clinical risk factor analysis in this manuscript. Total number of complete cases = 1091, total number of incomplete cases = 1985.OR; odds ratio, RF; rheumatoid factor, ERA; enthesitis related arthritis.


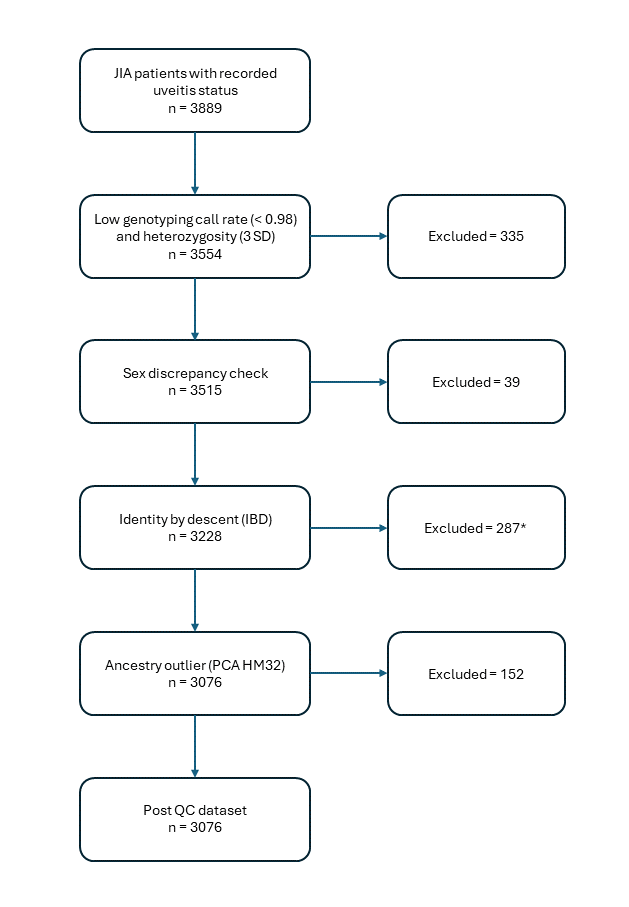


**Supplementary Figure 1:** quality control (QC) of individual level genotype data illustrating the exclusions made at each sample-level QC threshold. Exclude 1- Total of 355 individuals excluded due to low genotype call rate (<98%) and heterozygosity (3 SD). Exclude 2- Total of 39 individuals excluded due to discrepancy between genetically inferred sex and recorded sex. Exclude 3- Total of 287 individuals excluded by IBD indicating high levels of relatedness. Exclude 4- Total of 152 individuals excluded by PCA and the detection of ancestral outliers. JIA: juvenile idiopathic arthritis, SD: standard deviations, PCA: principal component analysis, HM3: HapMap 3. *IBD exclusions reflect participants recruited to multiple studies under different patient nomenclature schemes.

**
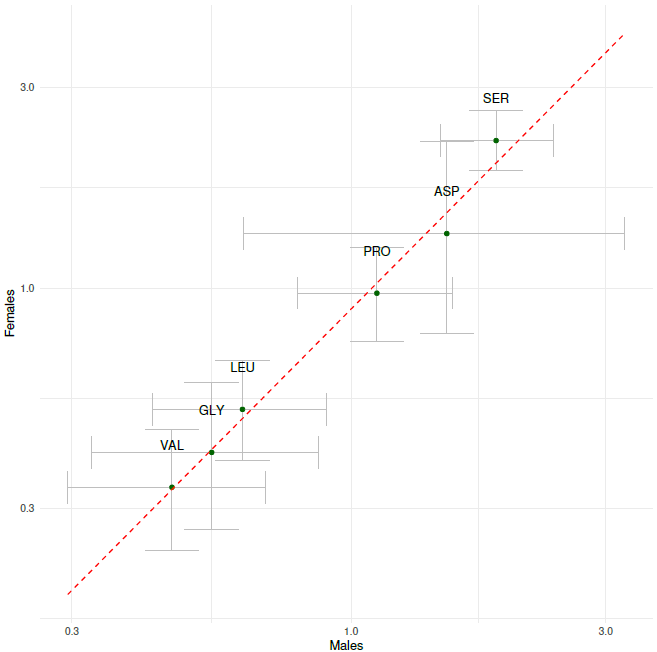
**

**Supplementary Figure 2:** Effect sizes (ORs and 95% CIs) of each residue at position 11 of *HLA-DRB1* in male and female cohorts. Ser; serine, Asp; aspartate, Pro; proline, Leu; leucine, Gly; glycine, Val; valine. The effect sizes of each residue in females (vertical axis) is plotted against the effect sizes of each residue in males (horizontal axis). Error bars represent the 95% confidence interval

**Genetic factors refine risk in JIA ILAR subgroups**: supplementary analysis of oligoarticular and rheumatoid factor negative polyarticular JIA

It is difficult to clinically differentiate between oligoarthritis and RF negative polyarthritis subtypes, therefore a further supplementary analysis was completed, restricted to individuals recorded as oligoarthritis and RF negative polyarthritis. This group will be referred to as “polygos” for the remainder of this manuscript. The polygo group consisted of 2073 individuals: 479 with uveitis and 1594 without uveitis. Association testing on all *HLA* alleles in the polygo group revealed that *HLA-DRB1* position 11 presented the most risk to uveitis onset (*P* = 3.9x10^-23^). Furthermore, Serine at position 11 of *HLA-DRB1* suggested the most risk to uveitis at this position (OR 1.9, 95% CI 1.6-2.2).
